# Pharmacological targeting of apelin impairs glioblastoma growth

**DOI:** 10.1093/brain/awx253

**Published:** 2017-10-03

**Authors:** Elizabeth Harford-Wright, Gwennan Andre-Gregoire, Kathryn A Jacobs, Lucas Treps, Sophie Le Gonidec, Heloise M Leclair, Sara Gonzalez-Diest, Quentin Roux, François Guillonneau, Delphine Loussouarn, Lisa Oliver, François M Vallette, Fabienne Foufelle, Philippe Valet, Anthony P Davenport, Robert C Glen, Nicolas Bidere, Julie Gavard

**Affiliations:** 1 CRCINA, Inserm, Team SOAP, CNRS, Universite de Nantes, Nantes, France; 2 Institut Cochin, Team SOAP, Inserm, CNRS, Universite Paris Descartes, Paris, France; 3 I2MC, Inserm, Universite Paul Sabatier, Toulouse, France; 4 3P5 Proteomics Facility of the Universite Paris Descartes, Paris, France; 5 Centre Hospitalier Universitaire (CHU) de Nantes, Nantes, France; 6 CRCINA, Inserm, Universite de Nantes, Nantes, France; 7 Institut de Cancérologie de l’Ouest, René Gauducheau, St Herblain, France; 8 Centre de Recherches des Cordeliers, Inserm, Universite Paris Descartes, Paris, France; 9 Experimental Medicine and Immunotherapeutics, University of Cambridge, Cambridge, UK; 10 The Centre for Molecular Informatics, Department of Chemistry, University of Cambridge, Cambridge, UK; 11 Computational and Systems Medicine, Department of Surgery and Cancer, Faculty of Medicine, Imperial College London, UK

**Keywords:** glioblastoma initiating cells, vascular niche, apelin, APJ, antagonist

## Abstract

Glioblastoma are highly aggressive brain tumours that are associated with an extremely poor prognosis. Within these tumours exists a subpopulation of highly plastic self-renewing cancer cells that retain the ability to expand *ex vivo* as tumourspheres, induce tumour growth in mice, and have been implicated in radio- and chemo-resistance. Although their identity and fate are regulated by external cues emanating from endothelial cells, the nature of such signals remains unknown. Here, we used a mass spectrometry proteomic approach to characterize the factors released by brain endothelial cells. We report the identification of the vasoactive peptide apelin as a central regulator for endothelial-mediated maintenance of glioblastoma patient-derived cells with stem-like properties. Genetic and pharmacological targeting of apelin cognate receptor abrogates apelin- and endothelial-mediated expansion of glioblastoma patient-derived cells with stem-like properties *in vitro* and suppresses tumour growth *in vivo*. Functionally, selective competitive antagonists of apelin receptor were shown to be safe and effective in reducing tumour expansion and lengthening the survival of intracranially xenografted mice. Therefore, the apelin/apelin receptor signalling nexus may operate as a paracrine signal that sustains tumour cell expansion and progression, suggesting that apelin is a druggable factor in glioblastoma.

## Teaser

Glioblastomas are aggressive brain tumours that contain a subpopulation of highly plastic self-renewing cancer cells. Harford-Wright *et al.* show that the vasoactive peptide apelin, secreted by brain endothelial cells, regulates glioblastoma patient-derived cells with stem-like properties. Pharmacological blockade of apelin hampers glioblastoma cell expansion and improves survival in xenografted mice.

## Introduction

Glioblastoma is the most common and lethal primary brain tumour in adults. Although there has been notable progress in strategies to fight glioblastoma (Stupp *et al.*, 2009; Chinot *et al.*, 2014; Brown *et al.*, 2016), the prognosis remains extremely poor with average survival reported to be less than 15 months following diagnosis (Stupp *et al.*, 2009, 2015). A subpopulation of tumorigenic cells termed glioblastoma stem-like cells (GSCs), also known as cancer-initiating cells (Lathia *et al.*, 2015), has been implicated in tumour initiation, resistance to current therapies and disease recurrence (Singh *et al.*, 2004; Bao *et al.*, 2006; Chen *et al.*, 2012; Yan *et al.*, 2013). Similar to how normal stem and progenitor cells participate in tissue development and repair, cancer stem-like cells pervert these processes to facilitate the initiation and progression of tumours. Moreover, GSCs contribute to both radiation and chemo-resistance as these treatments target cycling, highly proliferative cancer cells, whereas GSCs are comparatively quiescent, and thus survive to repopulate the tumour post-treatment (Bao *et al.*, 2006; Chen *et al.*, 2012). As such, GSCs represent an important target for future therapies and a better understanding of how GSCs interact with their environment is required.

Studies have proposed that GSC tumorigenicity relies on the surrounding tumour microenvironment, with brain tumour-initiating cells reported to reside in close contact with brain microvascular cells (Calabrese *et al.*, 2007; Galan-Moya *et al.*, 2011; Shingu *et al.*, 2017). The localization of GSCs in proximity to endothelial cells facilitates communication between these cells (Calabrese *et al.*, 2007) allowing the tumour vascular bed to provide factors essential to maintain GSC resistance to therapies, identity and fate (Garcia-Barros *et al.*, 2003; Folkins *et al.*, 2007; Evers *et al.*, 2010; Galan-Moya *et al.*, 2011, 2014). Among the putative candidates of this angiocrine signalling, soluble growth factors emanating from the vascular niche have been reported in various physiological and pathological models (Andreu-Agullo *et al.*, 2009; Beck *et al.*, 2011; Cao *et al.*, 2014, 2017). However, to date, the specific endothelial secreted factors involved in this process remain to be identified. Here, we used a mass spectrometry proteomic analysis of the endothelial cell secretome and identified the vasoactive peptide apelin as a central regulator of the expansion of glioblastoma patient-derived cells with stem-like properties. As such, targeting apelin may represent an effective novel therapeutic approach to treat glioblastoma.

## Materials and methods

### Ethics statement

Informed consent was obtained from all patients prior to sample collection for diagnostic purposes. Clinical tissue samples were provided by the Regional Institute for Cancer in Nantes Atlantique (IRCNA) tumour library (Nantes, France). This study was reviewed and approved by the institutional review boards of Sainte Anne Hospital, Paris, France, and Laennec Hospital, Nantes, France, and performed in accordance with the Declaration of Helsinki Protocol. Animal procedures were conducted as outlined by the European Convention for the Protection of Vertebrate Animals used for Experimental and other Scientific Purposes (ETS 123) and approved by the French Government (APAFIS#2016-2015092917067009).

### Analysis of human clinical databases

The Cancer Genome Atlas (TCGA, HG-UG133A and Agilent-4502A data), Rembrandt and Gravendeel microarrays were interrogated through the Gliovis platform (http://gliovis.bioinfo.cnio.es/) (Bowman *et al.*, 2017). Data were plotted based on histology criteria only. For reverse protein phase arrays (RPPA), optimal cut-offs were set to define high versus low expression of *APLNR*, as indicated on the plots. Pairwise *t*-tests were run.

### Cell culture, conditioned media preparation and mass spectrometry

Glioblastoma patient-derived cells with stem-like properties (GSCs) were isolated as previously described (Treps *et al.*, 2016). Briefly, tumours were dissociated using the MACsDissociator (Miltenyi) and each GSC characterized for their self-renewal capabilities, cell surface antigens, expression of stemness markers, their ability to differentiate, and to initiate tumour formation (Supplementary Table 1). GSCs 1–16 were maintained as spheres in NS medium (DMEM-F12, with N2, G5 and B27 supplements, GlutaMAX™ and antibiotics, Life Technologies). To induce differentiation in GSCs, the three supplements were omitted and 10% foetal bovine serum added to the media.

Human brain microvascular endothelial cells (hCMEC/D3, PO Couraud), HEK-293T and SVEC4-10 mouse endothelial cells (ATCC) were cultured as previously described (Treps *et al.*, 2016). Tumour-derived endothelial cells (tEC) were isolated from mechanically homogenized mice orthotopic brain tumours using CD31 MicroBeads (Miltenyi).

Stealth non-silencing control (low-GC 12935111) and selected siRNA targeting human *APLN* (HSS113086), *APLNR* (HSS100325) and *GSK3B* (HSS104522) (Life Technologies, 50 nM) were transfected using RNAiMAX Lipofectamine® (Life Technologies). GIPZ lentiviral shRNAs against human *APLNR* sequences 1–3, with identification numbers V3LHS_307344, V3LHS_307345 and V3LHS_307346, respectively, were purchased from Thermo Fisher Scientific. Lentiviral particles were collected from pGIPZ, pSPAX2 and pVSVg co-transfected HEK-293T cells (Dubois *et al.*, 2014).

Conditioned media (CM) from hCMEC/D3 (hEC-CM), tumour xenograft-derived endothelial cells (tEC-CM), SVEC4-10 (mEC) and HEK-293T (293T-CM) cells were obtained from 72-h-old monolayers in serum-free EBM2 (Lonza). Conditioned media from GSC#1 was obtained from 72-h-old tumourspheres. For acidic stress simulation, EBM2 (Lonza, pH 8.2) was adjusted to pH 6.8 using HCl before preparing hEC-CM. Apelin concentrations were quantified using the human apelin-12 EIA kit according to the manufacturer’s instructions (cross-reactivity with apelin-12, apelin-13, and apelin-36, Phoenix Pharmaceuticals).

Protein and peptide identification was performed in the University Paris Descartes Proteomics Facility (3P5, Paris, France), without trypsin proteolysis for peptidome analysis, as previously described in Luissint *et al.* (2012). Mass spectra were measured with a 4800 MALDI-TOF-TOF mass spectrometer (ABSciex) equipped with a Nd:YAG pulsed laser (355 nm wavelength, <500 ps pulse and 200 Hz repetition rate). Spectra acquisition and processing were performed using the 4000 series explorer software (ABSciex).

### Drugs

MM54 (cyclo[1-6]CRPRLCKHcyclo[9-14]CRPRLC) and MM193 were prepared as previously described (Macaluso *et al.*, 2011). Temozolomide (TMZ) and tideglusib were purchased from Sigma, and apelin peptides were from Phoenix Pharmaceuticals (pyr1-apelin-13 pyr1-QRPRLSHKGPMPF, apelin-13 QRPRLSHKGPMPF, and apelin-36 LVQPRGSRNGPGPWQGGRRKFRRQRPRLSHKGPMPF).

### Tumoursphere formation

To test the tumoursphere formation, GSCs (100 cells/µl) were plated in triplicate in indicated media as previously described (Harford-Wright *et al.*, 2016). Cells were manually dissociated each day and a single cell suspension maintained until Day 5. Tumourspheres were counted in five random fields of view, and the mean from the triplicate of each condition calculated from three independent experiments.

### Limiting dilution assays

To test the clonal capacity of GSCs, a limiting dilution assay was performed as previously described (Tropepe *et al.*, 1999). GSCs were seeded in the tested media (NS, MF and EC-CM) in a 96-well plate with serial dilutions ranging from 4 to 2000 cells/well, with eight wells per dilution for each plate and treated as indicated. Two weeks later, each well was scored for tumoursphere formation and the frequency of stem cells calculated using ELDA software (Hu and Smyth, 2009). The mean stem cell frequency for each condition was determined by averaging the stem cell frequencies of two independent experiments.

### Radioligand binding and calcium mobilization assays

Radioligand binding and calcium mobilization assays to assess the putative off-target effects of MM54 were performed by Eurofins Cerep Panlabs, according to the manufacturer's instructions.

### Cell viability

Cell viability in response to MM54 was tested using the UptiBlue reagent (Interchim), a fluorometric/colorimetric growth indicator based on the detection of metabolic activity. Briefly, cells were seeded at a density of 2 × 10^3^ per well, UptiBlue added at a concentration of 10% v/v and cells maintained at 37°C 5% CO_2_ until analysis. Absorbance was measured at Day 5 following treatment at 570 and 600 nm on a FLUOStar OPTIMA (BMG Labtech) plate reader, and the percentage of cell viability calculated according to the manufacturer’s instructions.

Cell survival in adherent cells was evaluated using the MTT assay [1-(4,5-dimethylthiazol-2-yl)-3,5-diphenylformazan, thiazolyl blue formazan; Sigma], which is reduced to formazan based on the mitochondrial activity of living cells. Cells were seeded in a 96-well plate in triplicate at a density of 5 × 10^3^ per well and treatments administered 24 h after seeding. Absorbance values were read at 590 nm and expressed as a percentage of cell viability relative to basal conditions.

### Animal procedures

Tumour inoculation experiments were performed on female Balb/C nude mice (Janvier) aged 5–6 weeks. For toxicity experiments 6-week-old female C57/Bl6J (Janvier) mice were used. Animals were randomly assigned to each group and group housed in specific pathogen-free conditions at 24°C on a 12-h day-night cycle. At all times, animals were allowed access to standard rodent pellets and water *ad libitum*.

To test potential toxic effects of MM54 and MM193 *in vivo*, mice were administered 2 mg/kg of MM54, MM193 or vehicle bi-weekly for 4 weeks. At sacrifice, blood was taken for analysis and the heart, kidney, aorta and liver removed, weighed and fixed for histological analysis. For the glycaemic study, animals were starved for 6 h prior to sacrifice.

For the ectopic models, mice were subcutaneously injected with 5 × 10^5^ GSC#9 in 100 μl of phosphate-buffered saline (PBS) and growth factor-free Matrigel® (Corning) in each flank. Tumourspheres were dissociated prior to injection for all *in vivo* experiments to ensure implantation of a single cell suspension. To analyse tumour initiation, mice were examined weekly to monitor tumour growth and sacrificed between 6 and 7 weeks following implantation. For pharmacological studies, mice were treated twice per week once tumours were palpable, with MM54 (2 mg/kg), MM193 (2 mg/kg) or vehicle (PBS) by intraperitoneal injection. Tumour size was measured once a week with callipers and tumour volume calculated using the following equation (width^2^ × length)/2.

Intracranial injection of GSC#9 was performed using a free hand injection technique as described in detail elsewhere (Treps *et al.*, 2016). Briefly, mice were anaesthetized with a mixture of ketamine (100 mg/kg) and xylazine (10 mg/kg) and a midline incision performed. A small burr hole was made 2 mm to the right of bregma, 1 mm anterior and 3 mm ventral to the coronal suture. A 5 μl Hamilton syringe was inserted to a depth of 3 mm and 10^5^ GSC#9 injected slowly. One minute after completion of the injection, the needle was retracted, surgical site closed and animals allowed to recover. At 3 weeks following GSC#9 inoculation, treatment with PBS or MM54 (2 mg/kg) was commenced three times per week until death due to tumour burden or the conclusion of the experiment at Day 70.

### Immunostaining

Both cellular and tissue analysis was performed using immunostaining and haematoxylin and eosin standard protocols (Treps *et al.*, 2016). The following primary antibodies were used: PECAM (BD), pS9-GSK3β (Cell Signaling), APLN and APLNR (Abcam), and Ki67, SOX2 and NESTIN (Millipore). Cell death was estimated through the TUNEL assay kit (Trevigen). A minimum of three tumour sections per condition was used for analysis, with at least five different fields of view. For blood vessel surface analysis, PECAM pixel intensity was calculated (ImageJ) in randomly chosen fields of view and mean ± standard error of the mean (SEM) of the total field of view was represented. Cell proliferation was assessed through the percentage of Ki67-positive cells normalized to the total number of nuclei. NESTIN-positive and pS9-GSK3β-positive cells were counted per field of view. Image acquisitions were performed on Spinning Disk Leica microscope (Institut Cochin) and confocal Nikon A1 RSi (Micropicell).

### Flow cytometry

For cell surface expression analysis, cells were incubated with antibodies for 1 h and washed twice with cold PBS. For total expression, cells were fixed (4% paraformaldehyde-PBS, 15 min) and permeabilized (iced-cold methanol, 10 min) prior incubation with antibodies. APC-APLNR, and isotype control Ig (R&D systems) antibodies were used.

Analysis of aldehyde dehydrogenase (ALDH) activity was performed using the ALDEFLUOR™ assay kit (Stem Cell Technologies). Briefly, cells were incubated with ALDEFLUOR alone or in combination with an ALDH activity inhibitor (DAEB) at 37°C for 45 min. This flow cytometry-based staining allows monitoring ALDH activity in stem, progenitor and cancer precursor cells. The ALDH activity is considered positive in comparison to cells incubated with DEAB reagent.

Flow cytometry analyses were performed on Accuri C6 and FACsCalibur (BD Biosciences, Cytocell) and processed using CFlow plus or FlowJo software (BD Biosciences).

### Western blots

Following stimulation with the relevant treatment, cells were collected and washed in PBS before lysis at 4°C with TNT buffer (50 mM Tris pH 7.4, 150 mM NaCl, 2 mM EDTA, 1% Triton™ X-100, 1% IGEPAL®) supplemented with protease inhibitors (ThermoFisher Scientific). Equal amounts of protein were loaded on tris-glycine gels and transferred onto nitrocellulose membranes (GE Healthcare). Antibodies against pS9-GSK3β, GSK3β, KDM1A, pS473-AKT, AKT, pS235/S236-S6 and pT202/Y204-ERK1/2 (Cell Signaling, Ozyme), GAPDH (Santa Cruz Biotech) and APLNR (Abcam) were incubated with the membrane overnight at 4°C and followed by incubation with the relevant secondary antibodies (Southern Biotech) for 1 h at room temperature. Membranes were revealed using a chemiluminescent HRP substrate (Millipore) and visualized using the Fusion imaging system (Vilber Lourmat).

### RNA extraction and RT-PCR

RNA was extracted using the Qiagen RNeasy® Mini Kit as per the manufacturer’s directions. Equal amounts of RNA were reverse transcribed using the SuperScript® III RT kit (Life Technologies) and the resulting cDNA was used to amplify mRNA by PCR using gene-specific primer sets in the presence of REDTaq® DNA polymerase (Sigma). *ACTB* and *GAPDH* were also amplified as control for input. See Supplementary Table 2 for primer details.

### Statistics

Data are representative of three independent experiments, unless otherwise stated. Statistical analysis was performed with GraphPad Prism6 using two-way ANOVA and an unpaired two-tailed *t*-test (Student’s *t*-test). In Kaplan-Meier survival curves, differences were compared by log-rank analysis and Gehan-Breslow-Wilcoxon. In all experiments a *P*-value of <0.05 was considered significant.

## Results

### Endothelial cells produce the vasoactive peptide apelin

**Figure 1:**
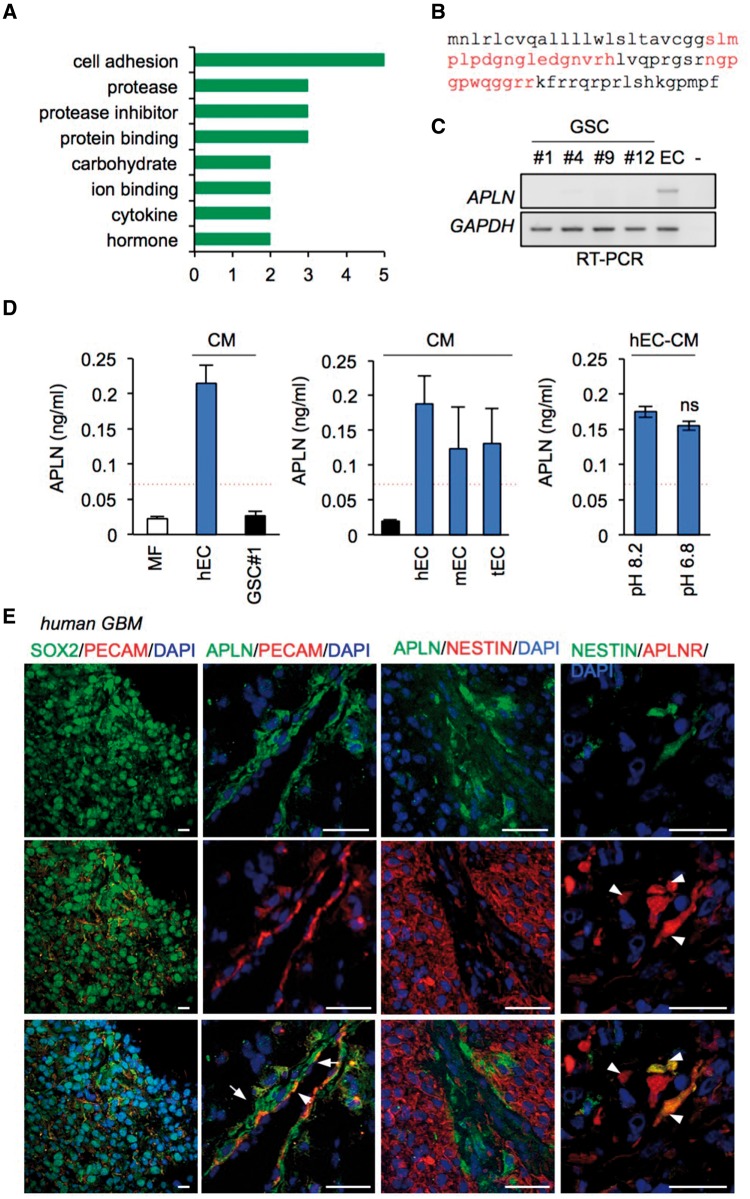
**Endothelial cells produce the vasoactive peptide apelin.** (**A**) Mass spectrometry analysis of the brain microvascular endothelial cell (hEC) secretome identified 22 peptides and proteins specific to endothelial cells. (**B**) Apelin peptide coverage (37%) is indicated in red on the full-length sequence. (**C**) RT-PCR for *APLN* and *GAPDH* is shown for hEC and glioblastoma patient-derived cells with stem properties (GSCs) #1, #4, #9 and #12 RNA total cell lysates. (**D**) Apelin secretion in mitogen-free control media (MF), and in conditioned media (CM) prepared from GSC#1, human brain microvascular EC (hEC), mouse macrovascular EC (mEC) and orthotopic mouse brain tumour-isolated EC (tEC). Apelin secretion was measured in CM from hEC cultured in acidified medium (pH 6.8) or control conditions (pH 8.2). Data are representative of *n* ≥ 2 with mean ± SEM. Red dashed lines indicate the minimum sensitivity range of APLN detection. (**E**) Confocal analysis of SOX2 (green) + PECAM (red), APLN (green) + PECAM (red), APLN (green) + NESTIN (red), NESTIN (green) + APLNR (red) in glioblastoma clinical samples. Nuclei are shown in blue (DAPI). Arrowheads and arrows indicate APLNR/NESTIN and APLN/PECAM-double positive cells respectively. Scale bars = 25 µm. Data are representative of *n = *4 newly diagnosed patient samples. All panels are representative of *n = *3, unless specified.

To identify endothelial-secreted factors potentially involved in the maintenance of GSCs, we performed an unbiased tandem mass spectrometry proteomic analysis of the human brain endothelial secretome using human brain endothelial cell (hCMEC/D3)-conditioned media (EC-CM) and compared it to epithelial-like HEK-293T CM. Hits that were shared by the two cell lines were discarded, and 22 peptides or proteins specific to the EC-CM identified (Fig. 1A, Supplementary Table 3 and Supplementary Fig. 1A). Apelin peptides revealed the highest exponentially modified protein index and were selected for further characterization (Fig. 1B, Supplementary Table 3 and Supplementary Fig. 1B and C).

Enzyme immunoassay analysis demonstrated that endothelial cells secreted significant amounts of apelin, as the peptide was robustly detected in the conditioned media produced by human, mouse and xenograft tumour-derived endothelial cells, supporting endothelial cells as a source of apelin (Fig. 1C and D). In contrast, apelin was not detected in patient-derived GSC#1, #2, #9 and #12 RNA lysates, and concentrations were found lower than the limit of ELISA sensitivity (0.07 ng/ml) (Fig. 1C and D). Furthermore, to challenge apelin production in conditions that recapitulate the tumour microenvironment, we assessed apelin secretion from human brain endothelial cells under acidic stress (Fig. 1D). Interestingly, acidification of the milieu did not affect the overall production of apelin.

Moreover, we detected apelin and its receptor, the G-protein coupled receptor APLNR (APJ), in clinical glioblastoma samples in the vicinity of PECAM-labelled endothelial cells and cells positive for the stem cell markers NESTIN and SOX2 (Fig. 1E), suggesting a potential role for apelin in the tumour vascular niche (Calabrese *et al.*, 2007). However, APLN staining did not coincide with NESTIN-positive tumour cells, but rather with vascular tracks (Fig. 1E), supporting endothelial cells as a potential source for apelin in glioblastoma, consistent with a recent report in colorectal cancer-derived endothelial cells (Zuurbier *et al.*, 2017). To explore the clinical relevance of apelin further, we performed a retrospective analysis using The Cancer Genome Atlas (TCGA), Rembrandt and Gravendeel databases. Analysis of all three databases revealed a significant increase in *APLN* mRNA in glioblastoma tissue, as compared to non-tumour samples, which might be due to endothelial abundance in these grade IV tumours (Supplementary Fig. 1D).

### Apelin sustains GSC expansion *in vitro*

**Figure 2:**
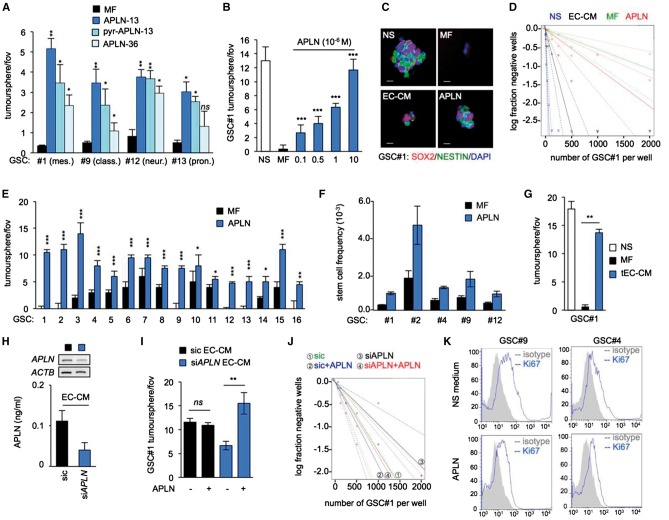
**Apelin sustains GSC expansion *in vitro*.** (**A**) Tumoursphere per field of view (fov) in GSCs #1, #9, #12 and #13 in response to apelin 13 (APLN-13), pyr-apelin-13 (pyr-APLN-13) or apelin 36 (APLN-36) treatment (1 μM, diluted in mitogen-free medium, MF). **P < 0.01, *P < 0.05 compared to the MF condition. (**B**) Tumourspheres per field of view were counted in GSC#1 cultured in complete mitogen-supplemented medium (NS), MF and MF supplemented with the indicated APLN concentration. ***P < 0.001 compared to the MF condition. (**C**) Confocal analysis of NESTIN (green), SOX2 (red) and nuclei (DAPI, blue) in GSC#1 grown in NS, MF, human brain endothelial cell-conditioned medium (EC-CM) or MF+APLN (1 μM). Scale bars = 20 µm. (**D**) Linear regression plot of *in vitro* limiting dilution assay (LDA) for GSC#1 in NS, EC-CM, MF, and MF+APLN (1 μM). Data are representative of *n = *2. (**E**) Tumourspheres per field of view were quantified in GSCs #1 to #16 cultured in MF or with apelin. *P < 0.05; **P < 0.01; ***P < 0.001 compared to the MF condition. (**F**) Stem cell frequency in GSCs #1, #2, #4, #9 and #12 in response to MF and APLN conditions. (**G**) Tumourspheres per field of view in GSC #1 in NS, MF and EC-CM derived from mouse tumour endothelial cells (tEC-EM). **P < 0.01 compared to the MF condition. (**H**) EC received non-silencing RNA (sic) or siRNA targeting APLN (si*APLN*) and APLN knockdown efficiency assessed by RT-PCR and ELISA. (**I** and **J**) GSCs #1 were cultured with sic and si*APLN* EC-CM, with or without apelin (1 μM). **P < 0.01 compared to the corresponding control condition for both tumoursphere and LDA assays. (**K**) FACS analysis of the proliferation marker Ki67 in GSCs #4 and #9 in NS and MF+APLN conditions. All panels are representative of *n = *3, unless otherwise specified.

We next evaluated the response of patient-derived GSCs, which have been extensively characterized both *in vitro* and *in vivo* (Supplementary Table 1 and Supplementary Fig. 2A and B) to the biologically active apelin fragments: apelin-13, pyr-apelin-13 and apelin-36 (see ‘Materials and methods’ section for more information). Although all of the apelin peptides increased the number of tumourspheres compared to mitogen-free media (MF), apelin-13 was the most potent at sustaining GSCs (Fig. 2A). Subsequently, we assessed the effect of increasing concentrations of apelin-13 (termed apelin hereafter) on GSC#1 and observed a potent and sustained increase in tumourspheres from the lowest concentration (Fig. 2B). Consistent with our previous work (Galan-Moya *et al.*, 2011, 2014), both mitogen-supplemented medium (NS) and EC-CM maintained the expression of stem markers NESTIN and SOX2 (Fig. 2C). Accordingly, mitogen withdrawal resulted in the loss of expression of these markers and the reduced ability to form tumourspheres, which was rescued by the addition of synthetic apelin to this MF media (Fig. 2C). To determine whether apelin alone maintained GSC self-renewal, a limiting dilution assay was performed in GSC#1 (Fig. 2D). As expected, we observed the highest frequency of colony-forming cells in GSCs grown in NS and EC-CM. Nonetheless, compared to MF conditions, GSC#1 grown in apelin-supplemented MF demonstrated an increase in the frequency of colony-forming cells*.* Moreover, we observed in a panel of 16 patient-derived GSCs (Supplementary Table 1) that apelin-supplemented media significantly increased the ability of GSCs to expand as tumourspheres (Fig. 2E), and increased the frequency of stem cells in a panel of five representative GSCs (Fig. 2F), indicating that *in vitro* apelin addition sustains GSC growth and substitutes, at least partially, to cell culture supplements provided in the NS (Fig. 2D–F). Similar effects were obtained with apelin-containing conditioned media derived from mouse brain tumour endothelial cells (tEC-CM) (Figs 1C and 2G), indicating that tumour-derived endothelial cells may provide a source of bioactive apelin *in situ*, although the intratumoural concentration and the apelin forms are not experimentally available. Consistent with these findings, EC-CM obtained from *APLN*-silenced endothelial cells was no longer able to maintain the stem properties of GSCs, while the addition of exogenous apelin into the depleted EC-CM restored this effect (Fig. 2H–J). Furthermore, we did not observe any obvious effect of apelin-supplemented mitogen-free media on the proliferation of GSCs (Fig. 2K), indicating that apelin may maintain GSCs by enhancing their self-renewal capabilities.

### Apelin modulates GSCs via activation of the G-protein coupled receptor APLNR

**Figure 3:**
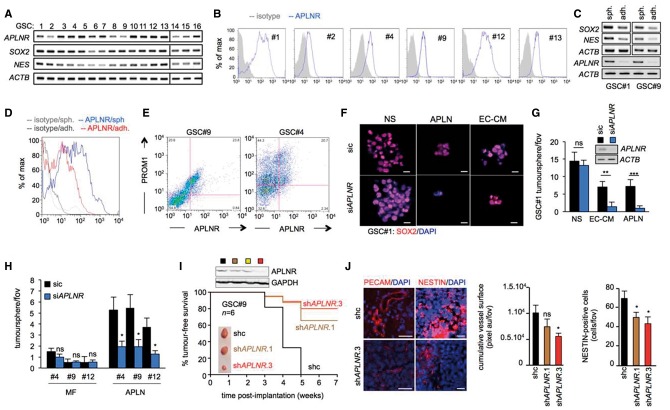
**Apelin modulates GSCs via activation of the G-protein coupled receptor APLNR.** (**A**) RT-PCR in a panel of 16 GSCs for *APLNR* and stemness markers *NES* and *SOX2*. *ACTB* is shown as internal PCR control. (**B**) FACS analysis of APLNR surface expression in GSCs #1, #4, #9, #12 and #13. (**C** and **D**) Differentiation was induced in GSCs #1 and #9 by growth in serum-containing media. RT-PCR and FACS analysis of APLNR and stem markers in GSCs #1 and #9 grown as both tumourspheres (sph.) and differentiated adherent cells (adh.). (**E**) FACS analysis of the stemness marker PROM1 (CD133) and APLNR in GSCs #4 and #9. Data are representative of *n = *2. (**F**) GSC #1 received non-silencing RNA (sic) or *APLNR* targeting siRNA (si*APLNR*) and were maintained in complete medium (NS), human brain endothelial cell-conditioned medium (EC-CM), and MF supplemented with purified apelin (APLN, 1 μM). Confocal analysis of SOX2 (red) and nuclei (DAPI, blue). Scale bars = 20 μm. (**G**) Tumourspheres per field of view (fov) in sic (small interfering control) or si*APLNR* GSC#1 maintained in NS, EC-CM or APLN. *APLNR* knockdown was assessed with RT-PCR. **P < 0.01; ***P < 0.001 compared to the sic condition. (**H**) Tumourspheres per field of view in non-silencing duplexes (sic) or *APLNR* targeting siRNA (si*APLNR*) transfected GSC#4, #9 and #12 in MF alone or supplemented with APLN. (**I**) GSCs#9 were infected with control shRNA (shc, black), and shRNA targeting *APLNR (*seq#1, orange; seq#2, yellow; and seq#3, red). Knockdown efficiency was checked by western blots. Female nude mice were implanted with 5 × 10^5^ shcontrol (black line), sh*APLNR* seq#1 (orange line) or seq#3 (red line) and monitored for tumour-free survival over 7 weeks. *n = *4 mice/group. (**J**) Sections of tumour tissue were analysed for PECAM and NESTIN expression using immunofluorescence. Scale bar = 40 μm. *n* ≥ 4 mice/group. All panels are representative of *n = *3, unless specified.

Apelin is known to signal through the G-protein coupled receptor APLNR (also known as APJ), which is reported to be highly expressed throughout the brain and act as paracrine and autocrine factor that supports embryonic and tumour angiogenesis (Kaelin *et al.*, 2007). In the present study, we observed a heterogeneous expression of APLNR in our panel of GSCs, at both a RNA and protein level (Fig. 3A and B). In keeping with a role for apelin in the stem cell maintenance, we found that differentiated GSCs were associated with a decrease in APLNR expression compared to tumourspheres (Fig. 3C and D) and reduced tumour-initiating ability (Supplementary Fig. 2C). Moreover, analysis of the stem marker PROM1 (CD133) revealed that expression of APLNR was detected in the PROM1 (CD133)-positive GSC population, further supporting a role for apelin and its receptor in the stem population (Fig. 3E). Consistent with this, APLNR silencing in GSC#1 impaired the ability of these cells to form SOX2-positive spheres cultured in both EC-CM and apelin conditions (Fig. 3F and G). Of note, the optimal concentration of exogenous mitogens in the NS medium allows maintaining *APLNR*-knocked down GSC#1 expansion *in vitro* (Fig. 3F and G). Similar results were obtained in three additional GSCs with variable APLNR expression level (Fig. 3A, B and H), highlighting the potential importance of this receptor in GSC maintenance in response to APLN. Subsequently, GSC#9 was transduced with short hairpin (sh) RNA against *APLNR* and grafted subcutaneously into the flanks of nude mice. Reducing APLNR levels in GSC#9 markedly decreased tumour development, NESTIN overall staining and only mildly affect tumour vascularization (Fig. 3I and J). To observe the impact of APLNR signalling on tumour development in the brain microenvironment, shAPLNR GSC#9 were orthotopically implanted into the striatum of nude mice and assessed for histological signs of tumour growth at Week 5, when tumours are largely developed but neurological signs were not yet evident. In these conditions, the number of progressing tumours was modestly reduced in *APLNR* shRNA (Supplementary Fig. 2D). Whether the reduction of *APLNR* expression also decreases tumour volume would require in-depth measurement over time. This slight decrease in tumour formation suggested that APLNR contributes to tumour expansion, although compensatory mechanisms may take place due to alternate signalling or an incomplete knockout of *APLNR* gene. Collectively, these results suggest that endothelial-secreted apelin sustains GSCs both *in vitro* and *in vivo* via activation of the apelin receptor.

### Pharmacological inhibition of APLNR impairs the effects of the endothelial secretome on GSCs by inhibition of GSK3β signalling

**Figure 4:**
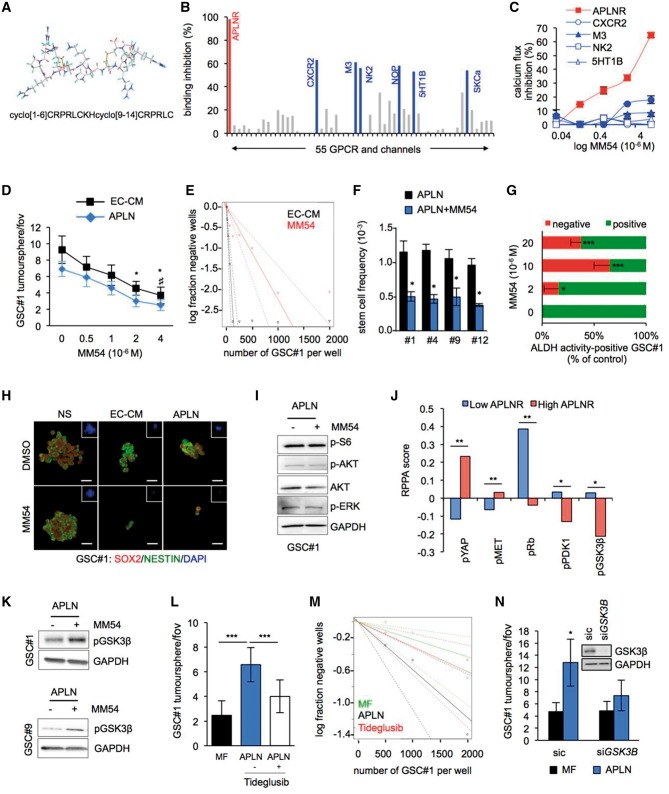
**Pharmacological inhibition of APLNR impairs the effects of the endothelial secretome on GSCs by inhibition of GSK3β signalling.** (**A**) Molecular structure and primary sequence of the competitive APLNR antagonist MM54. (**B**) A radioligand binding assay of 55 G-protein coupled receptors and ion channels identified APLNR (indicated in red) and six putative off-targets (indicated in blue) that demonstrated >50% inhibition of agonist binding following administration of APLNR antagonist MM54 (10 μM). (**C**) The percentage of calcium flux inhibition following MM54 treatment (0.4–10 μM) in the G-protein coupled receptor hits. (**D**) Tumoursphere per field of view (fov) in response to MM54 (0–4 μM) treatment in GSC#1 maintained in human brain endothelial cell-conditioned medium (EC-CM) and apelin-supplemented mitogen-free MF media (APLN, 1 μM) for 5 days. *P < 0.05 compared to EC-CM DMSO control, ^#^*P < *0.05 compared to apelin DMSO control. (**E**) Linear regression plot of *in vitro* limiting dilution assay (LDA) for GSC#1 in EC-CM or EC-CM + MM54 (2 μM). (**F**) Stem cell frequency in apelin supplemented media in response to MM54 (2 μM) in GSCs #1, #4, #9 and #12. *P < 0.05 compared to the vehicle condition. (**G**) Flow cytometry analysis of the percentage of ALDH positive and negative GSC #1 in response to 2, 10 or 20 μM of MM54 at Day 5. ALDH activity corresponds to the percentage of cells that contains ALDH activity (positive) or not (negative), normalized to the vehicle condition. *P < 0.05; ***P < 0.001 compared to the vehicle condition. (**H**) Confocal analysis of GSC #1 treated with DMSO or MM54 (2 μM) for SOX2 (red), NESTIN (green) and nuclei (DAPI, blue). Scale bars = 20 µm. (**I**) Western blot analysis of components of the mTOR and ERK signalling pathways in GSC#1 with APLN in the presence or absence of MM54 (2 μM). (**J**) Reverse protein phase array (RPPA) from the TCGA database were analysed in low and high *APLNR* expressing glioblastoma samples. *P < 0.05; **P < 0.01 compared to the low APLNR condition. (**K**) Western blot analysis of pS9-GSK3β in GSCs #1 and #9 following MM54 treatment in APLN containing MF media. (**L**) Tumoursphere per field of view in GSC#1 in response to APLN treatment (1 μM) in the presence or absence of the GSK3β inhibitor (tideglusib, 2.5 μM). ***P < 0.001 compared to the MF condition. (**M**) Linear regression plot of limiting dilution assay (LDA) for GSC #1 in MF and APLN (1 μM) alone or with tideglusib. (**N**) GSC #1 received sic (control) or *GSK3B* targeting siRNA (si*GSK3B*) and tumoursphere per field of view was quantified in MF supplemented with purified apelin (APLN, 1 μM). *P < 0.05 compared to the sic MF condition. All panels are representative of *n = *3, unless otherwise specified.

To next evaluate the potential of targeting apelin/APLNR, we investigated the properties of a novel bi-cyclic peptide [cyclo(1–6)CRPRLC-KH-cyclo(9–14)CRPRLC], MM54, which acts as a competitive antagonist of APLNR (Fig. 4A) (Macaluso *et al.*, 2011; Brame *et al.*, 2015). To identify possible off-target G-protein coupled receptor or ion channels that may interact with MM54, we performed radioligand competitive binding experiments to investigate the specificity of the compound. MM54 inhibited more than 95% of apelin binding to APLNR at the dose of 10 µM (Fig. 4B). In addition to APLNR, of the 55 receptors tested, five G-protein coupled receptors (CXCR2, M3, NK2, NOP, and 5HT1B) and one ion channel (SKCa) demonstrated over 50% inhibition of agonist binding in response to MM54 (10 μM) (Fig. 4B). However, using a cell-based second messenger assay to measure G-protein coupled receptor-mediated calcium flux we again observed that MM54 was very effective at inhibiting APLNR, while having little or no effect towards other identified off-targets (Fig. 4C). Thus, MM54 may behave as a potent and selective inhibitor of apelin binding and APLNR activation. In both EC-CM and apelin-supplemented mitogen-free (MF) media, MM54 induced a dose-dependent decrease in the number of tumourspheres that was significant from a concentration of 2 μM (Fig. 4D). In keeping with this, we observed a significant reduction in the frequency of sphere-forming cells in GSCs #1, #4, #9 and #12 following treatment with MM54 (Fig. 4E and F). Furthermore, inhibition of APLNR with MM54 clearly decreased the percentage of the stem marker aldehyde dehydrogenase (ALDH)-positive cells compared to untreated GSC#1 controls (Fig. 4G), consistent with the MM54-mediated decrease in the number of SOX2- and NESTIN-positive spheres (Fig. 4H). However, GSC#1 were resistant to MM54 treatment when cultured in mitogen-containing defined medium (NS) that does not contain apelin, consistent with our RNA interference data (Fig. 3). Analysis of downstream mechanisms associated with apelin/APLNR activation revealed that MM54 did not induce any changes to major components of the PI3K/AKT and ERK signalling pathways (Fig. 4I). To explore the APLNR downstream signalling further, we interrogated the TCGA database for reverse phase protein array (RPPA) in glioblastoma patients with high and low APLNR expression (Fig. 4J). This analysis unmasks two significantly upregulated phospho-proteins, namely pYAP and pMET, and three downregulated (pRb, pPDK1, and pGSK3β) in high APLNR glioblastoma samples (Fig. 4J). Interestingly, glycogen synthase kinase 3β (GSK3β) activity has recently been shown to participate in gliomagenesis via maintenance of the stem population of cancer cells (Zhou *et al.*, 2016). This process occurs through the GSK3β-dependent stabilization of KDM1A. Moreover, GSK3β inactivation by phosphorylation on serine 9 was associated with a loss of stemness traits in GSCs (Zhou *et al.*, 2016). In keeping with this, incubation with MM54 (2 μM, overnight) in apelin-supplemented MF media resulted in an increase in phosphorylation of GSK3β at serine 9 in both GSCs #1 and #9 (Fig. 4K), consistent with an inhibitory effect on GSK3β signalling. Consequently, we treated patient-derived GSCs with the GSK3β inhibitor tideglusib (2.5 μM) and observed that apelin was less potent at increasing tumourspheres and self-renewal (Fig. 4L and M). Furthermore, silencing GSK3β in GSC#1 resulted in a significant decrease in apelin-mediated tumoursphere formation (Fig. 4N), suggesting that apelin may sustain GSCs via activation of GSK3β signalling.

### Pharmacological inhibition of APLNR by MM54 impairs the *in vitro* expansion of temozolomide-resistant GSCs

**Figure 5:**
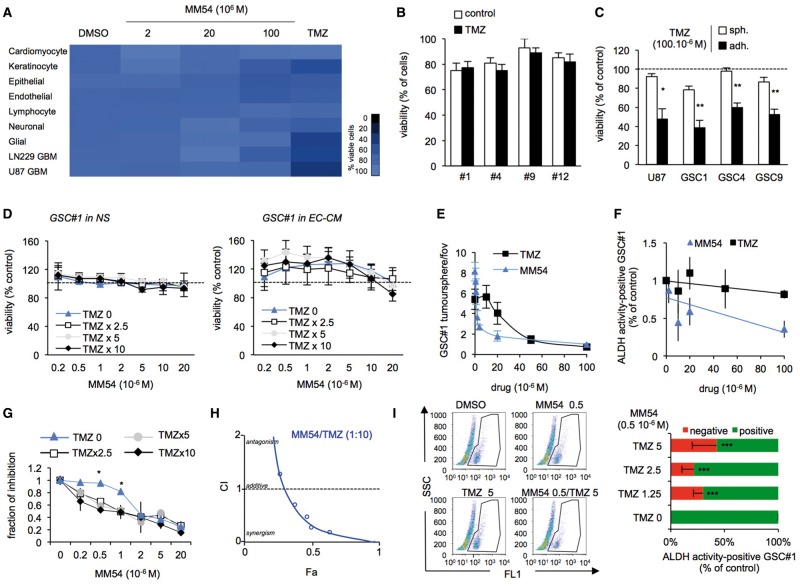
**Pharmacological inhibition of APLNR by MM54 impairs the *in vitro* expansion of temozolomide-resistant GSCs.** (**A**) Cell viability following treatment with DMSO, MM54 (2, 20 and 100 μM) or temozolomide (TMZ, 50 μM) was measured using UptiBlue in different cell types for 3 days. Cardiomyocytes (mouse primary cardiomyocytes), keratinocytes (HaCAT), epithelial cells (CaCo2), endothelial (hCMEC/D3), lymphocyte (Jurkat), neuronal (SH-SY5Y), glial (SVGp12). (**B** and **C**) Cell viability following treatment with DMSO or TMZ (100 μM) was measured using UptiBlue in GSCs #1, #4, #9, and #12 for 3 days. Similar experiments were conducted U87 glioblastoma cell line and GSCs #1, #4, and #9 grown as spheroids (sph.) in NS medium or as differentiated adherent cells (adh.) in serum-containing medium. (**D**) GSC#1 viability was assessed following combined treatment with MM54 (0.2–20 μM) and TMZ (constant ratios TMZ:MM54 2.5:1, 5:1, and 10:1) in NS and human brain endothelial cell-conditioned medium (EC-CM) conditions. (**E** and **F**) Tumoursphere per field of view (fov) and ALDH activity were assessed in response to MM54 (0.2–100 μM) or TMZ (10–100 μM) treatment at Day 5. (**G**) Drugs were combined at a constant MM54:TMZ ratio (1:2.5, 1:5, and 1:10) and ALDH activity measured. *P < 0.05 compared to the TMZ 0 condition. (**H**) Combination index plot for TMZ with MM54. Combination index (CI) was plotted against fractions affected (Fa) and analysed using COMPUSYN (http://www.combosyn.com/). A result <1 indicated an additive effect of the two compounds, while values closer to 0 suggest the drugs may behave synergistically. (**I**) Flow cytometry analysis of ALDH activity in GSC #1 at Day 5 following combined treatment with MM54 (0.5 μM) and the indicated TMZ doses. ALDH activity corresponds to the percentage of cells that contains ALDH activity (positive) or not (negative). ***P < 0.001 compared to the TMZ 0 condition. All panels are representative of *n = *3, unless otherwise specified.

The chemotherapeutic agent TMZ is commonly used in the treatment of glioblastoma, although it has been reported that GSCs are resistant to TMZ (Chen *et al.*, 2012; Hale *et al.*, 2013). To test the specificity of MM54 towards GSCs, we treated a panel of normal human and primary glioblastoma cell lines with increasing concentrations of MM54, as compared to TMZ. MM54 demonstrated no overtly toxic effects on any of the cell lines tested, whilst TMZ significantly reduced the viability of glioblastoma cell lines (namely U87 and LN229) but not GSCs (Fig. 5A and B). Similarly to GSC#1, #4, #9 and #12, the *in vitro* viability of U87 glioblastoma cell line grown as spheroids was not modified upon high dose of TMZ (Fig. 5C). Conversely, TMZ reduces the viability of U87 glioblastoma cell line and GSCs #1, #4 and #9, when grown as adherent differentiated cells (Fig. 5C).

Combined treatment of MM54 with TMZ did not significantly alter GSC#1 viability in NS or EC-CM even at the highest concentrations of both compounds (Fig. 5D). Moreover, MM54 was significantly better at impairing GSC#1 tumoursphere formation and ALDH activity at low doses compared to TMZ, which required much higher concentrations to achieve comparable results (Fig. 5E and F). Drugs were then combined at constant MM54:TMZ ratios (1:2.5, 1:5, and 1:10) and ALDH activity measured (Fig. 5E). At MM54 suboptimal dose, i.e. <2 μM MM54 (Fig. 4), TMZ significantly potentiates the effects of MM54. To further assess whether MM54 and TMZ do synergize, data were processed according to the Chou combination index (CI) method (Chou, 2010) (Fig. 5G). In this representation, a CI value of 1 indicates an additive effect, <1 synergism and >1 antagonism. TMZ and MM54 therefore displayed a striking synergism (Fig. 5H). In line with this, co-administration of low doses of both MM54 (0.5 μM) and TMZ (1.25, 2.5, and 5 μM) decreased the percentage of ALDH activity in GSCs (Fig. 5I), indicating that APLNR antagonists may enhance the therapeutic efficacy of TMZ.

### Pharmacological inhibition of APLNR by MM54 reduces xenograft progression

**Figure 6:**
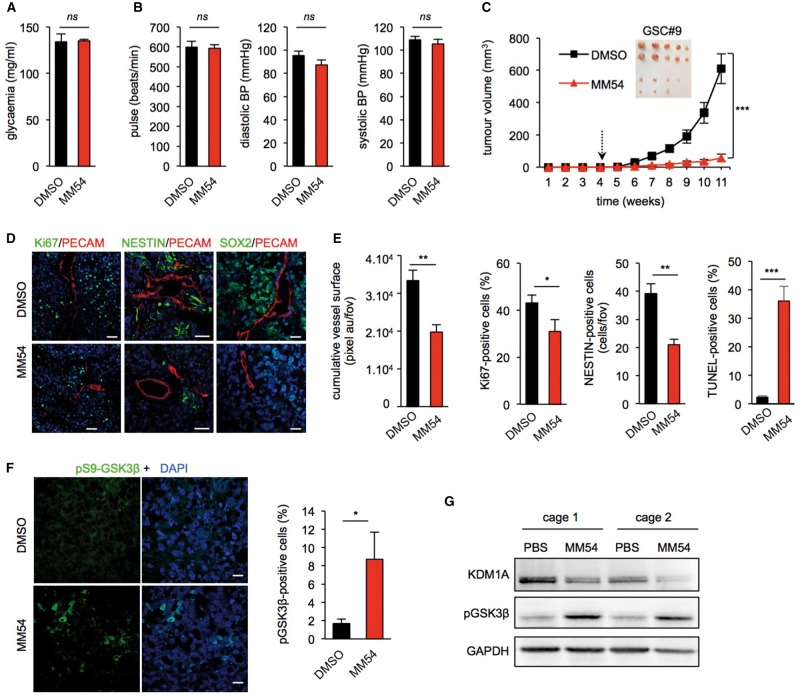
**Pharmacological inhibition of APLNR by MM54 reduces xenograft progression.** (**A**) Tumour-bearing mice were fasted for 6 h and the effect of either MM54 (2 mg/kg) or DMSO vehicle treatment on glycaemia measured via blood analysis. (**B**) Cardiac frequency and blood pressure were measured in random-fed tumour-bearing animals. (**C**) Nude mice were implanted with GSC#9 (5 × 10^5^ cells) in each flank and treated with either DMSO vehicle or the APLNR antagonist (MM54, 2 mg/kg) bi-weekly from Week 4. Tumour volume was measured weekly until Week 11. *n = *10/group. (**D** and **E**) Cryosections from GSC tumours were assessed for PECAM (red), Ki67 (green), NESTIN (green), SOX2 (green) and apoptosis (TUNEL). (**F**) Tumour sections were assessed for pS9-GSK3β staining in DMSO vehicle- and MM54-treated animals. Scale bars = 40 µm. (**G**) Western blot analysis of KDM1A and pS9-GSK3β was performed on two independent tumours from each treatment group. *n = *6 mice/group. *P < 0.05; **P < 0.01; ***P < 0.001 compared to the DMSO vehicle control group. All panels are representative of *n = *3, unless specified.

Pharmacodynamics studies revealed that MM54 demonstrated good solubility in the tested solutions, and was detected in the plasma and the brain *in vivo* following intraperitoneal administration in healthy animals (Supplementary Table 4). Next, to determine the bio-safety of MM54 *in vivo,* tumour-bearing mice were administered 2 mg/kg of MM54 bi-weekly for 4 weeks. Due to the known physiological roles of apelin on the cardiovascular system and glucose metabolism (Maguire *et al.*, 2009; Scimia *et al.*, 2012; Fournel *et al.*, 2015), cardiac frequency, blood pressure and glycaemic index were measured. MM54 did not induce alterations to these parameters, reflecting no obvious detrimental action of APLNR antagonism in tumour-bearing animals (Fig. 6A and B). Complete blood count analysis revealed no significant differences between mice treated with MM54 and vehicle in healthy animals (Supplementary Table 5). Similarly, histological and biochemical analysis of heart, kidney and liver revealed no differences between MM54-treated animals and vehicle controls (Supplementary Fig. 3), indicating that at the present dose following repeated administration, MM54 does not exert any overt adverse effects *in vivo*.

We next tested the effect of pharmacological inhibition of APLNR with MM54 in an ectopic xenograft tumour model. MM54 treatment dramatically reduced tumour growth over 11 weeks when compared to DMSO control group (Fig. 6C). The decreased tumour volume was associated with a reduction in the staining of SOX2 and NESTIN-positive cells, overall proliferation and viability that was accompanied by a diminution in tumour vascularization (Fig. 6D and E). Additionally, MM54 treatment led to a significant increase in phospho-GSK3β positive cells within the tumour (Fig. 6F and G). In line with Zhou *et al.*rsquo;s (2016) studies, this increased GSK3β phosphorylation was correlated with a decrease in KDM1A levels (Fig. 6G). To further validate our findings with MM54, we tested a second recently developed and structurally different APLNR antagonist, MM193 (Glen and Davenport, unpublished observation). Increasing doses of MM193 in GSCs counteracted the effect of apelin on tumourspheres *in vitro* (Supplementary Fig. 4A). Moreover, administration of MM193 (2 mg/kg) in GSC#9-inoculated mice resulted in significant impairment of tumour growth compared to vehicle controls (Supplementary Fig. 4B). Likewise, blockade of APLNR with MM193 did not induce any adverse changes to cardiac frequency, blood pressure or glycaemia in healthy animals (Supplementary Fig. 4C). Together, these *in vivo* data indicate that pharmacological inhibition of APLNR efficiently and safely reduces tumour growth in xenografted female animals.

### Pharmacological blockade of APLNR by MM54 prolongs survival of xenografted mice

**Figure 7:**
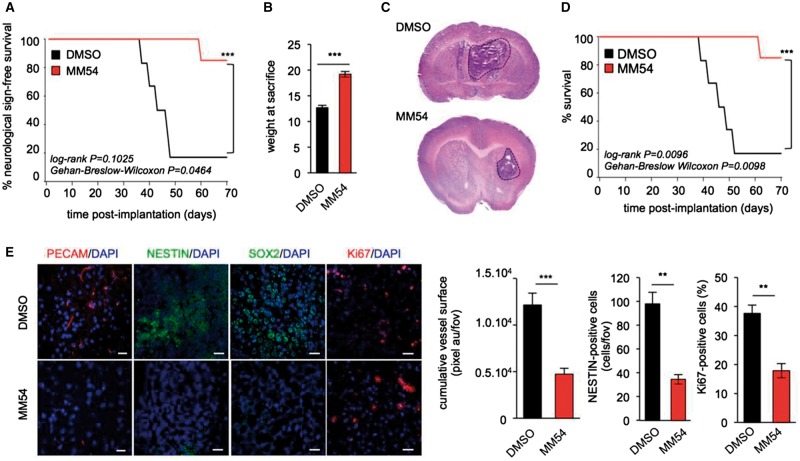
**Pharmacological blockade of APLNR by MM54 prolongs survival of xenografted mice.** (**A–E**) 10^5^ GSC #9 were implanted into the striatum of female nude mice and treated three times a week with DMSO or MM54 (2 mg/kg) from Week 3 and the appearance of neurological symptoms monitored over time (**A**). The weight of mice at sacrifice was recorded for each treatment group (**B**). Haematoxylin and eosin (H&E) staining of tumour-inoculated brains following MM54 (2 mg/kg) or DMSO vehicle treatment (**C**). Kaplan-Meier survival curve of GSC #9 bearing mice in response to vehicle or MM54 treatment. *n = *6/group. (**D**) Cryosections of brain tumour tissue stained for PECAM (red), NESTIN (green), SOX2 (green), Ki67 (red), and DAPI (blue) and quantified. Scale bars = 40 µm. **P < 0.01; ***P < 0.001 compared to the DMSO control group (**E**). All panels are representative of *n = *3, unless otherwise specified.

To gain further insight into the therapeutic potential of APLNR antagonism in glioblastoma, nude mice were orthotopically implanted with GSC#9 into the striatum and treated with MM54 (2 mg/kg) three times a week. Experimental models of brain tumours are commonly associated with the development of neurological symptoms as well as cachexia as the tumour progresses. MM54 treatment was sufficient to impair the development of tumour-associated neurological symptoms and weight loss (Fig. 7A and B), which was coupled with a marked reduction in tumour size (Fig. 7C). Importantly, MM54 administration significantly improved the overall survival of tumour-bearing mice compared to their vehicle-treated counterparts (Fig. 7D). Additionally, blockade of APLNR was associated with a reduction in vascularization, proliferation, and SOX2 and NESTIN-positive cells (Fig. 7E). Collectively, these *in vivo* data provide a strong basis for the clinical potential of apelin/APLNR signalling as a therapeutic target in glioblastoma.

## Discussion

The present study has identified the vasoactive peptide apelin as a critical factor involved in glioma growth. It is now well accepted that GSCs reside in proximity to vascular beds, into which endothelial cells secrete factors that regulate their self-renewal and fate. With that view, apelin is highly expressed in endothelial cells and once released has been proposed to act as a local mediator (Kleinz and Davenport, 2005; Kaelin *et al.*, 2007). In keeping with this, a recent study reports the high expression of apelin in colorectal cancer-isolated endothelial cells, which further correlates with refractoriness to anti-angiogenic treatment (Zuurbier *et al.*, 2017). Here, we demonstrate that apelin is released by human, mouse and tumour-derived endothelial cells *in vitro*, although this secretion was not overtly affected by the acidification of the milieu. Additionally, we show that apelin increases GSC self-renewal *in vitro* in tumoursphere and limiting dilution assays, and that this effect appears to be independent of cell proliferation, consistent with the previously reported action on microvascular endothelial cells (Kaelin *et al.*, 2007).

In both subcutaneous ectopic and intracranial orthotopic xenograft models, inhibition of APLNR was associated with a significant reduction in tumour volume together with a reduction in vascularization, proliferation and an increase in apoptosis. Moreover, animals implanted with *APLNR* knocked down cells (shAPLNR GSC#9) were associated with a reduction in tumour burden compared to control groups, indicating that APLNR may be intrinsically important for tumour development. Additionally, APLNR knockdown and MM54 treatment diminished the number of NESTIN-positive cells within the xenografts again strengthening our hypothesis that apelin is particularly essential for the maintenance of GSCs.

Moreover, apelin has been implicated in physiological and pathological angiogenesis (Kaelin *et al.*, 2007). Apelin induces proliferation and vessel sprouting in endothelial cells, as well as stabilizing contacts between adjacent endothelial cells (Kleinz and Davenport, 2005). In keeping with this, a recent study proposed apelin as a marker for monitoring tumour vessel normalization and response to anti-angiogenic therapy (Zhang *et al.*, 2016; Zuurbier *et al.*, 2017). Accordingly, pharmacological blockade of apelin (Figs 6, 7 and Supplementary Fig. 3), but not the reduction of APLNR expression in GSCs (Supplementary Fig. 2D), may also contribute to the reduction of tumour volume observed in this study *in vivo*, by blocking angiogenesis and depriving tumour cells of the nutrients they require to survive. Although we cannot discount alternative sources of apelin peptides are involved *in vivo*, taken together the results of this study indicate that endothelial-derived apelin is an important factor for glioma growth.

The poor response of glioblastoma to chemotherapies has been in part attributed to the population of resistant initiating cells within the tumour. Therefore, identification of agents that improve GSC sensitivity to TMZ, the current standard-of-care, is of great interest. It has been reported that vascular niche maintains GSCs in a quiescent state thereby protecting them from radiation and chemotherapies. Our study demonstrates that the APLNR antagonist MM54 synergizes with TMZ *in vitro*. We further demonstrate that TMZ alone does not alter the activity of the stem marker ALDH, however when combined with suboptimal dose of MM54, we observed profound alterations in the percentage of ALDH-positive cells. High *ALDH1A1* expression has been associated with poor prognosis in glioblastoma, and its overexpression *in vitro* a predictor of TMZ resistance (Schafer *et al.*, 2012). These alterations to the stem identity of GSCs suggest that combined treatment with MM54 and TMZ may provide an interesting opportunity to further target populations of cells currently resistant to chemotherapeutic drugs.

Although the precise molecular mechanisms that connect the apelin/APLNR axis to GSC maintenance will require further investigation, our data suggest that it may act through the GSK3β signalling pathway. GSK3β was shown to be upregulated in glioblastoma cells, and assist in stem cell maintenance by phosphorylating and stabilizing KDM1A (Zhou *et al.*, 2016). Paralleling the effect of the GSK3β inhibitor tideglusib (Zhou *et al.*, 2016) (Fig. 4L), we found that the APLNR antagonist MM54 reduced GSC self-renewal and potentiated sensitivity to TMZ (Fig. 5). APLNR inhibition was accompanied by an increased phosphorylation of GSK3β at S9, both *in vitro* and *in vivo* further supporting an inhibitory effect of MM54 compound on GSK3β signalling.

Here, we provide evidence that both *in vitro* and *in vivo* inhibition of APLNR results in a significant reduction in tumour growth. Given the concerns about the current therapeutic regime and the intrinsic resistance to TMZ, targeting apelin signalling presents a new opportunity for use in the treatment of glioblastoma.

## Supplementary Material

Supplementary Figures

Supplementary Tables

## Abbreviations

EC-CM: endothelial cell conditioned media
GSC: glioblastoma stem-like cells
MF: mitogen-free
NS: mitogen-supplemented
TMZ: temozolomide
