# Supplementary material for: Pharmacological targeting of apelin impairs glioblastoma growth
Source: Brain. 2017 Oct 3;140(11):2939–54. doi: 10.1093/brain/awx253 (PMC5841205; doi:10.1093/brain/awx253)
Supplement: Supplementary Figures [file brain-2017-00147-file009_awx253.pdf]

A

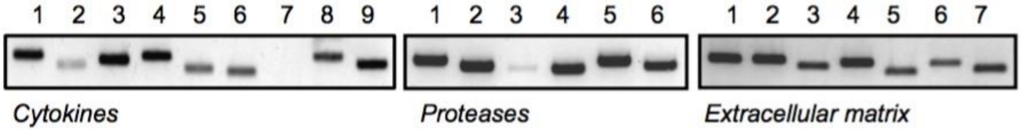

B

Start-End	Mass	ppm	Score	Expectation	Peptide
24-41	1920.918	-2.88	96	7.9e-08	G.SLMPLPDGNGLEDGNVRH.L
26-41	1720.813	3.46	69	1.8e-05	L.MPLPDGNGLEDGNVRH.L
26-41	1736.808	3.19	25	0.029	L.MPLPDGNGLEDGNVRH.L
28-41	1492.716	1.28	43	0.0012	P.LPDGNGLEDGNVRH.L
33-41	996.500	14.1	28	0.2	N.GLEDGNVRH.L
50-59	1025.499	7.36	52	0.0012	R.NGPGPWQGGR.R
50-60	1181.598	4.60	28	0.13	R.NGPGPWQGGRR.K
51-59	911.460	13.0	48	0.0099	N.GPGPWQGGR.R

C

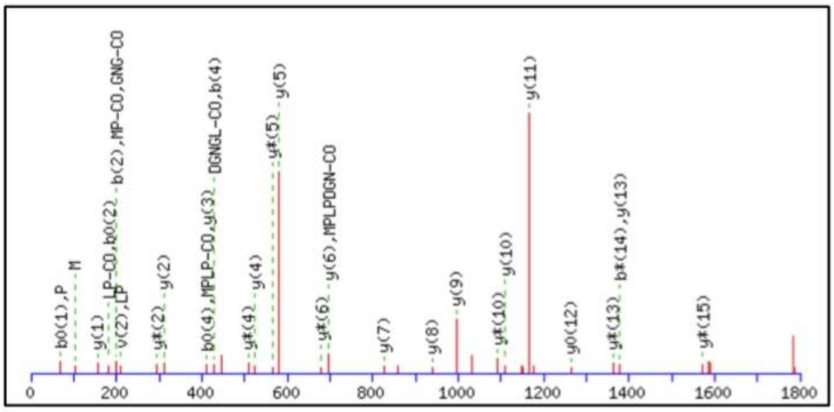

D

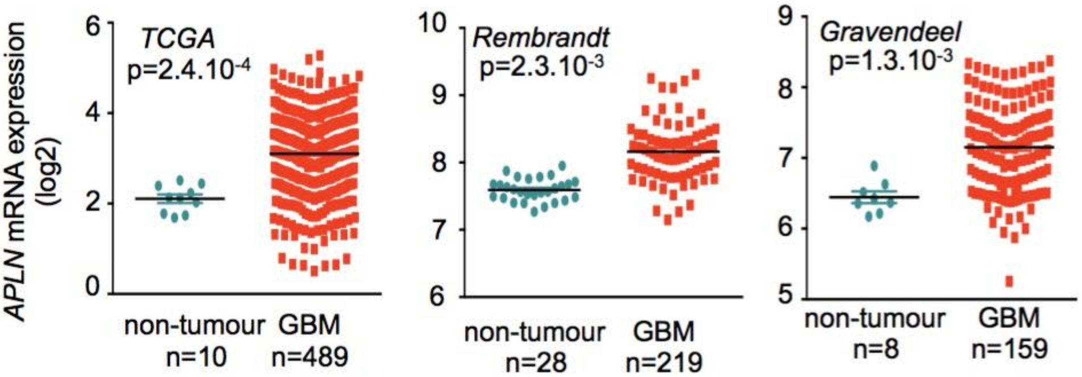

Figure S1. Apelin production by brain endothelial cells. (**A**) RT-PCR was used to check for the presence of the mRNA corresponding to the hits identified in the MS screen. Cytokines: 1. *ADM*, 2. *APLN*, 3. *CTGF*, 4. *FSTL1*, 5. *PTX3*, 6. *IGFBP7*, 7. *MIF*, 8. *TGFB2* , 9. *LGALS1;* Proteases: 1. *CST3*, 2. *SERPINE1*, 3. *TIMP1*, 4. *PRSS23*, 5. *CTSB*, 6. *SRGN;* Extracellular matrix: 1. *LGALS3BP*, 2. *FN1*, 3. *THBS1*, 4. *EFEMP1*, 5. *HSPG2*, 6. *LAMA5*, 7. *EDIL3*. (**B**) Peptide sequences corresponding to apelin found in MS are indicated. Mass: observed mass; ppm: parts per million; Expectation: number of matches with equal or better scores that are expected to occur by chance alone. (**C**) Peptide View. MS/MS fragmentation of **SLMPLPDGNGLEDGNVRH** sequence. (**D**) *APLN* mRNA expression in non-tumour and glioblastoma tissue using the TCGA, Rembrandt and Gravendeel databases. Data were analysed via the Gliovis platform (https://gliovis.shinyapps.io/GlioVis/).

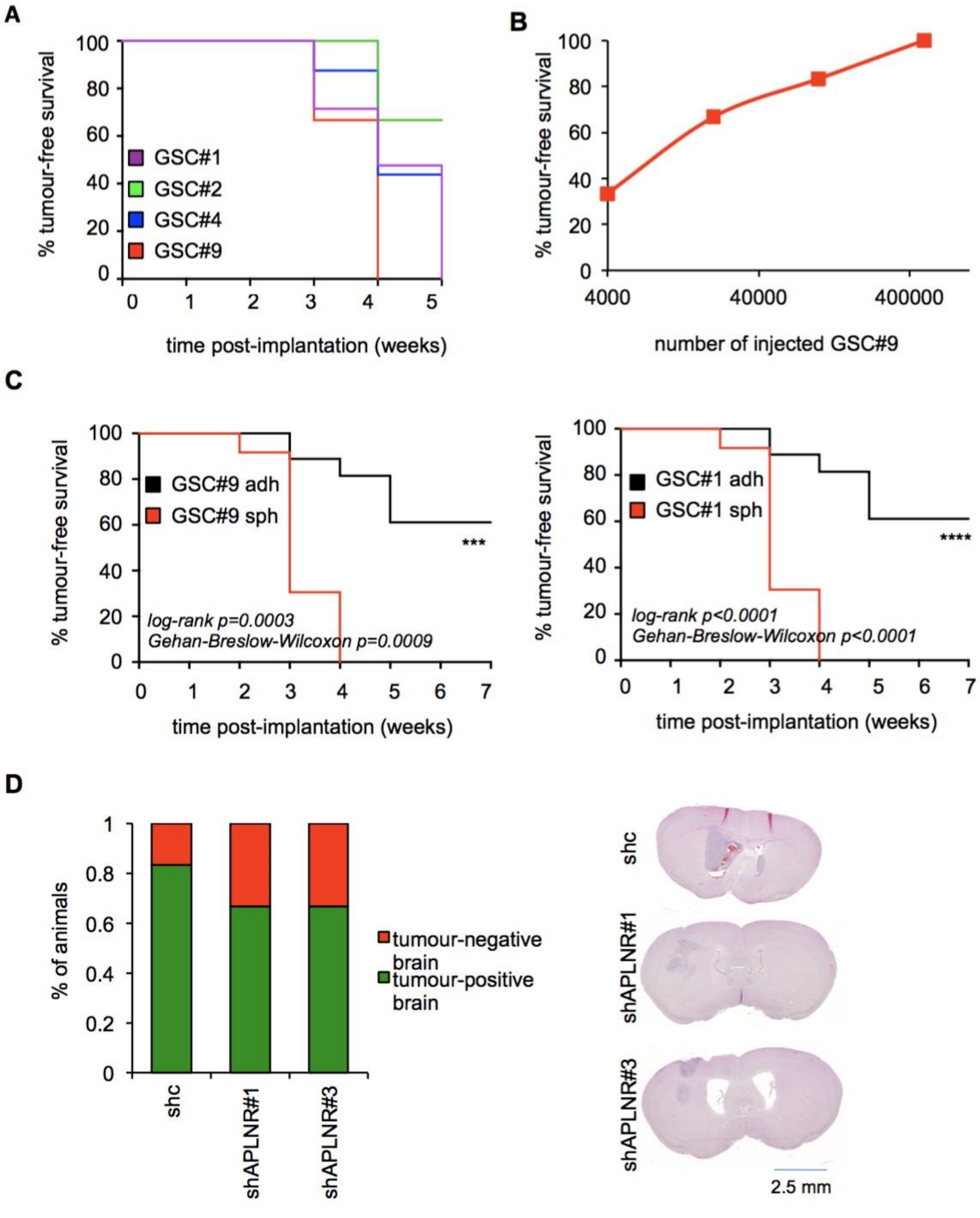

Figure S2. ***In vivo*** **characterisation of patient-derived glioblastoma cells with stem properties (GSC).** (**A**) 5.10^5 patient-derived Glioblastoma cells with stem properties (GSCs #1, #2, #4 and #9) were implanted in flanks of nude mice and number of non-regressing palpable tumours was scored over time. n=4. (**B**) Different amount of GSC#9 were implanted in flanks of nude mice and number of non-regressing palpable tumours was scored at week5. n=6. (**C**) 5.10^5 GSC#9 and GSC#1 cultured either as sphere (sph.) or adherent and differentiated (adh.) were implanted in flanks of nude mice and number of non-regressing palpable tumours was scored over time. n=6. (**D**) GSCs#9 were infected with control shRNA (shc), and shRNA targeting APLNR (sequence #1 and sequence #2,) and 10^5 were implanted in female nude mice. Animals were sacrificed at week 5 post-surgery and the presence of tumours was determined by HE staining (absence, red; presence, green on the graph). n≥5 mice/group. All panels are representative of n=3, unless specified.

.

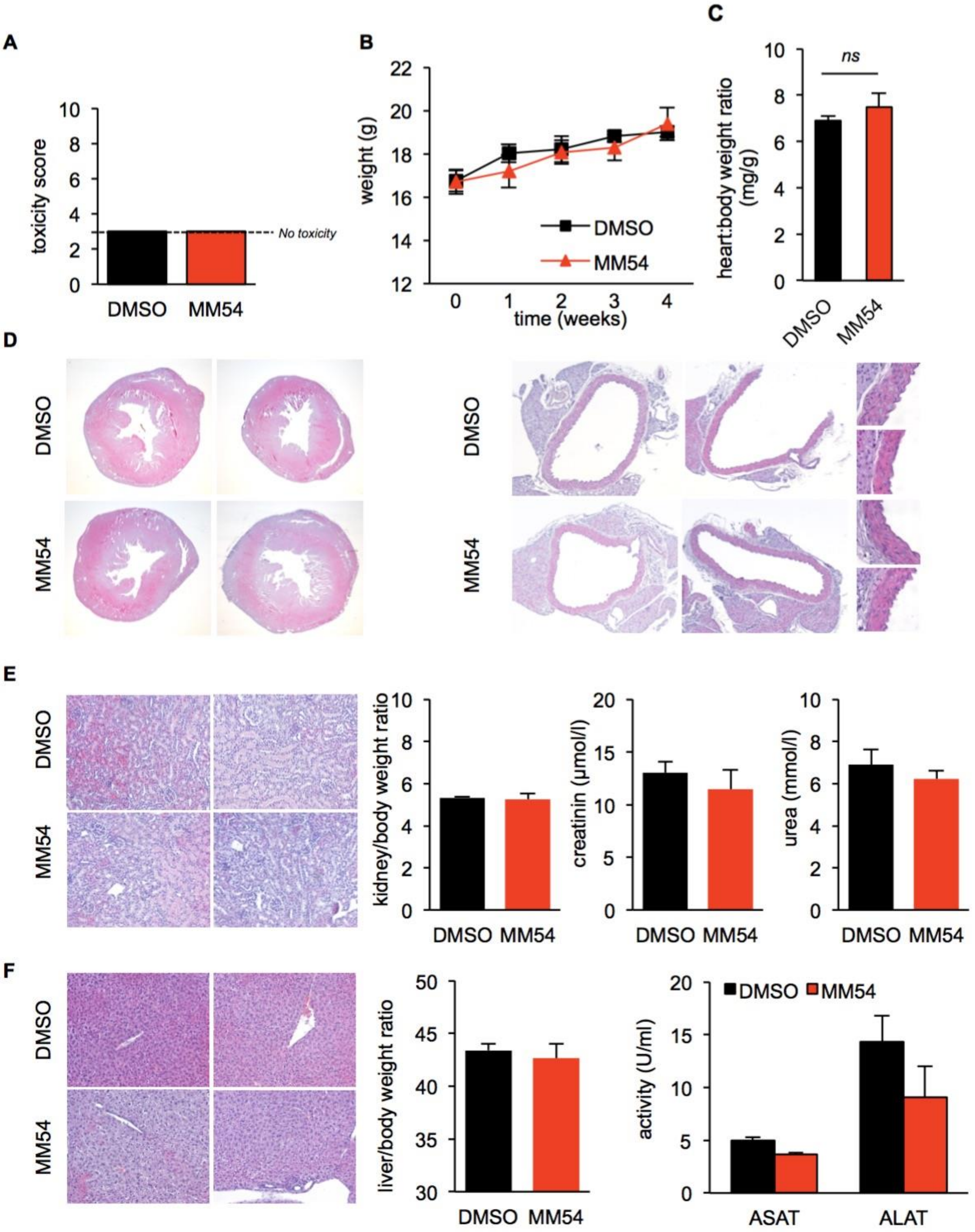

Figure S3. Pre-tolerance studies of MM54 in healthy mice. (**A**) C57Bl/6 mice were injected twice a week with MM54 and monitored for toxicity. (**B**) weight loss, (**C**) cardiac, (**D**) kidney and (**E**) liver function. n=4 mice/group, mean±SEM.

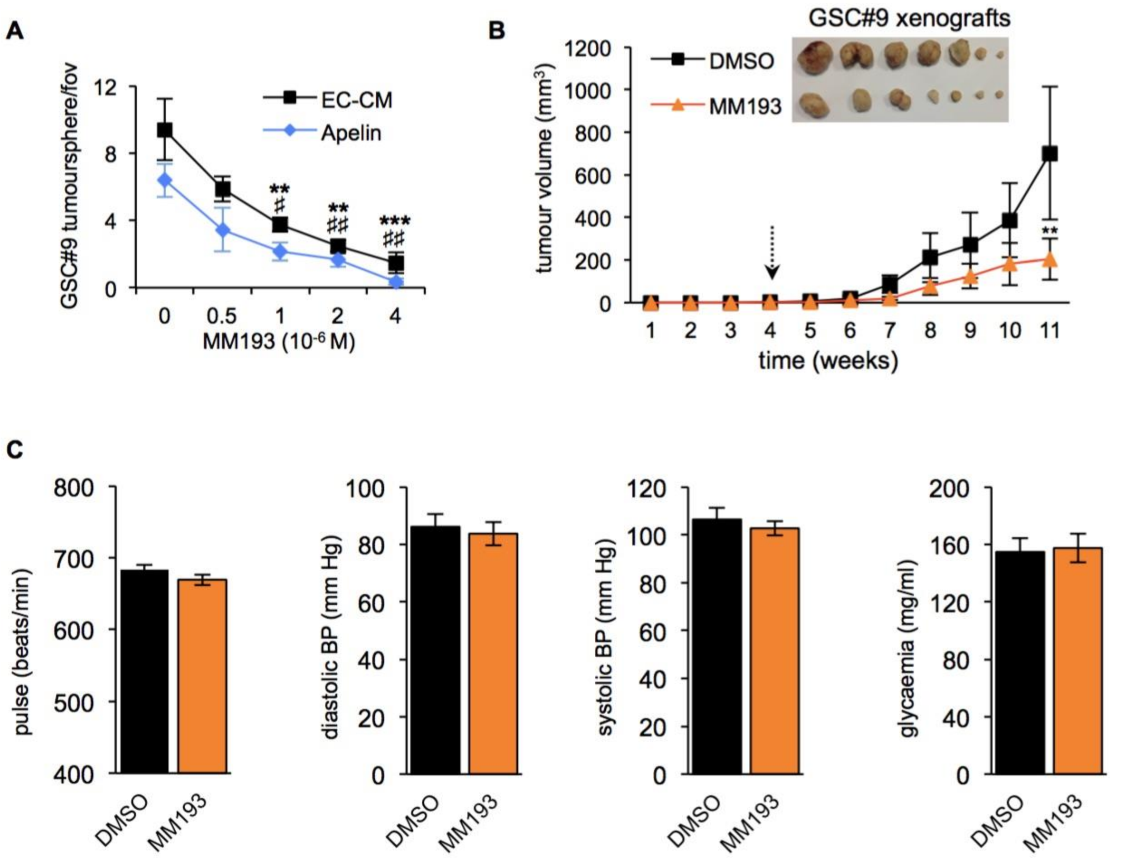

Figure S4. Efficacy of the APLNR antagonist MM193 in reducing xenograft progression. (**A**) Efficacy of the APLNR antagonist MM193 in reducing GSC#9 TS formation in response to increasing doses of the MM54 brother compound MM193 in endothelial cell conditioned medium (EC-CM) and apelin-supplemented mitogen-free media. n=3 mean±SEM. **p<0.01; ***p<0.001. (**B**) Nude mice were inoculated with GSC#9 and monitored for tumour growth following treatment with MM193. n=6 mice/group, mean±SEM. **p<0.01. (**C**) Analysis of cardiac frequency, blood pressure and glycaemic index in response to MM193 treatment in healthy C57Bl/6 mice. n=4 mice/group, mean±SEM.
