# Supplementary material for: Pharmacological targeting of apelin impairs glioblastoma growth
Source: Brain. 2017 Oct 3;140(11):2939–54. doi: 10.1093/brain/awx253 (PMC5841205; doi:10.1093/brain/awx253)
Supplement: Supplementary Tables [file brain-2017-00147-file010_awx253.pdf]

#	Age	Gender	Histology	Subtype (Verhaak)	Tumor Initiation	Differentiation	LDA
#1	68	M	GBM	Mes	+	+	8
#2	25	M	GBM	Mes	+	+	6
#3	67	M	GBM	Mes	n.d.	+	n.d.
#4	76	F	GBM	Mes	+	+	6
#5	66	M	GBM	Proneural	+	+	6
#6	68	M	GBM	Mes	+	+	1.6
#7	49	M	GBM	Mes	+	+	1.3
#8	79	M	GBM	Classic	+	+	6
#9	68	F	GBM	Classic	+	+	6
#10	N/A	M	GBM	Classic	+	+	2.6
#11	67	M	GBM	Proneural	+	+	4
#12	59	M	GBM	Neural	+	+	3
#13	73	F	GBM	Proneural	+	+	2.7
#14	70	F	GBM	Mes	+	+	2.4
#15	72	M	GBM	Classic	+	+	6
#16	65	F	GBM	Proneural	+	+	2.7

Table S1. GSC characterisation
LDA (limiting dilution assay) was measured as detailed in the method section. Differentiation was induced by switching cells from mitogen-containing media to DMEM supplemented with 10% FBS. Differentiation was considered as positive according to morphological changes of adherent cells within 10 days. Tumour initiation was assessed by intracranial implantation in nude mice and considered as positive when at least half of animals develop tumours within 3 months. n.d.: not determined.

Target	Left Primer	Right Primer
ACTB	GGACTTCGAGCAAGAGATGG	AGCACTGTGTTGGCGTACAG
ADM	CGTCGGAGTTTCGAAAGAAG	CCCTGGAAGTTGTTCATGCT
APLN	GGAAGTGCAGCAGGAATAGC	ACACACAAAGTTGGGCATCA
APLNR	GAGTGCTGGGAAGGACTCTG	ACTGGTTGTCTGCCCCATAG
CSTB	CCAGGGAGCAAGACAGAGAC	GAGACTGGCGTTCTCCAAAG
CST3	ACCAGCCACATCTGAAAAGG	AGAGGGGACAATCAGTGTGG
CTGF	GGAAAAGATTCCCACCCAAT	TGCTCCTAAAGCCACACCTT
EDIL3	CCCGAGGATTTAATGGGATT	GTGGGCCTGAGCATTTGTAT
EFEMP1	CAGGACACCGAAGAAACCAT	GTTTCCTGCTGAGGCTGTTC
FN1	ACCAACCTACGGATGACTCG	GCTCATCATCTGGCCATTTT
FSTL1	GCACAGGCAACTGTGAGAAA	CATAGTGTCCAAGGGCTGGT
HSPG2	CTGCCGTAATCTCCACCAAT	CTTTTGGCTGTGCAGATGAA
IGFBP7	AAGTAACTGGCTGGGTGCTG	TATAGCTCGGCACCTTCACC
LAMA5	TGACCTTTTCTGGCTCGTCT	GTTCAGCACAAAGGGCTCTC
LGALS1	CTCTCGGGTGGAGTCTTCTG	ACGAAGCTCTTAGCGTCAGG
LGALS3BP	ACCAATGAAACCAGGAGCAC	GCATCCACACTCATGGTGAC
MIF	GTTCCTCTCCGAGCTCACC	TGCTGTAGGAGCGGTTCTG
NES	AACAGCGACGGAGGTCTCTA	TTCTCTTGTCCCGCAGACTT
PRSS23	ACTTACGAAGAGGCCAAGCA	GTCCTTCCCAAAAATGCTGA
PTX3	GTGGGTGGAGAGGAGAACAA	TTCCTCCCTCAGGAACAATG
SERPINE1	CTCTCTCTGCCCTCACCAAC	GTGGAGAGGCTCTTGGTCTG
SOX2	ACACCAATCCCATCCACACT	GCAAACTTCCTGCAAAGCTC
SRGN	CAGGGTTTGAGGTTTTGGAA	CCGCGTAGGATAACCTTGAA
TGFB2	TGCTTTGGCTTTCTGGTTCT	TTTGTTTGTGGTGCAGTGGT
THBS1	AGGCATGTTCCAGTTTCACC	GCTGGCACCACCTTTATTGT
TIMP1	AATTCCGACCTCGTCATCAG	TGCAGTTTTCCAGCAATGAG

Table S2. RT-PCR primers

		Peptidome		Proteome	
Gene	Protein name	Score	emPAI	Score	emPAI
		**Cytokines**			
ADM	Adrenomedullin	483	24.02	-	-
APLN	Apelin	170	42.17	-	-
CTGF	Connective tissue growth factor	34	0.16	-	-
FSTL1	Follistatin-related protein 1	55	0.17	36	0.17
PTX3	Pentraxtin-related protein 3	86	0.51	124	0.91
IGFBP7	Insulin-like growth factor-binding protein 7	-	-	67	0.44
MIF	Macrophage migration inhibitory factor	-	-	50	0.53
TGFB2	Transforming growth factor beta-2	-	-	46	0.12
LGALS1	Galectin 1	-	-	49	0.43
		**Proteases**			
CST3	Cystatin C	110	1.05	89	0.97
SERPINE1	Plasminogen activator inhibitor 1	120	0.66	187	1.35
TIMP1	Metalloproteinase inhibitor 1	-	-	56	0.26
PRSS23	Serine protease 23	35	0.29	-	-
CTSB	Cathepsin B	-	-	36	0.15
SRGN	Serglycin	112	5.41	109	0.85
		**Extracellular matrix**			
LGALS3BP	Galectin-3-binding protein	150	0.7	268	0.65
FN1	Fibronectin	186	0.19	279	0.28
THBS1	Thrombospondin-1	182	0.56	271	0.65
EFEMP1	EGF-containing fibulin-like extracellular matrix protein-1	49	0.37	100	0.47
HSPG2	Basement membrane-specific heparan sulfate proteoglycan	-	-	46	0.04
	core protein	-	-	38	0.01
LAMA5	Laminin 5	-	-	47	0.11
EDIL3	EGF-like repeat and discoidin I-like domain-containing protein 3				

Table S3. Tandem mass spectrometry (MS/MS) endothelial cell secretome analysis
MS/MS analysis was performed on human brain endothelial cells (hCMEC/D3) conditioned media and compared to the HEK-293T one. Shared hits were removed from the list. Peptide mass tolerance was 20 and 30 parts per million for the peptidome and the proteome respectively, and the fragment mass tolerance was 0.3 Da for both experiments. The sum of the highest ions score for each distinct detected sequence (Score) and the exponentially modified protein abundance index (*em*PAI) are indicated for each of the 22 sequences identified either in the peptidome or proteome analysis.

A

Conditions	Solubility (mM)	Solubility ($mg.ml^{-1}$)
saline (NaCl 0.9%)	>0.73	>1.27
saline (15%) + kolliphor HS15	>0.69	>1.20
saline (10%) + kolliphor EL	>0.60	>1.04
saline (10%) + hydroxypropyl-β-cyclodextrin	>0.69	>1.20
saline (15%) + kolliphor HS15 + PEG (10%) + ethanol (5%)	>0.73	>1.27

B

Location	Plasma	Brain
**Half-life** (min)	142+/-22	n.d.
**AUC**	88391+/-731 ($min.ng.ml^{-1}$)	21633+/-2742 (min.ng)
**Time Cmax** (min)	10	10
**Cmax** ($\mu g.ml^{-1}$)<br>**Cmax** (μM)	1.1<br>0.6	0.26<br>0.14

Table S4. Pharmacodynamics of MM54 compound after intraperitoneal administration

A. MM54 compound (1 mg) was re-suspended in the different vehicles for 24h, RT, under gentle rotation. Solutions were analysed by HPLC and concentrations calculated using DMSO-re-suspended MM54 as standard. **B.** MM54 compound was solubilized in water and administered intraperitoneally (IP) at the dose of 3.6 mg/kg to healthy mice. Mice (3/group) were sacrificed at 10 min, 30 min, 60 min, 2h, 4h, 6h, 8h and 24h post-injection. Plasma and brains were collected and further processed for LC-MS/MS analysis. B/P index is 0.24. AUC is area under the curve. n.d. stands for 'not able to be determined'.

Total blood count	DMSO		MM54	
**Leukocytes**				
White blood cells (K/µl)	10,6	± 1,2	11,2	± 1,3
Neutrophils (K/µl)	3,07	± 0,29	2,56	± 0,66
Lymphocytes (K/µl)	5,90	± 0,86	7,50	± 0,51
Monocytes (K/µl)	0,47	± 0,06	0,42	± 0,11
Eosinophils (K/µl)	0,86	± 0,10	0,52	± 0,28
Basophils (K/µl)	0,31	± 0,04	0,19	± 0,12
**Erythrocytes**				
Red blood cells (M/µl)	6,8	± 0,9	7,5	± 0,3
Haemoglobin (g/dl)	8,9	± 1,2	9,9	± 0,6
Hematocrit (%)	31,8	± 4,3	35,4	± 1,9
Mean corpuscular volume (fl)	46,4	± 0,2	47,2	± 0,4
Mean corpuscular haemoglobin (pg)	13,0	± 0,0	13,2	± 0,2
Mean corpuscular haemoglobin concentration (g/dl)	27,9	± 0,1	27,9	± 0,3
Red cell distribution width (%)	15,7	± 0,2	16,1	± 0,1
**Thrombocytes**				
Platelet (K/µl)	314	± 28	301	± 27
Mean platelet volume (fl)	4,30	± 0,07	4,45	± 0,16

Table S5. Blood analysis after MM54 treatment
In order to test potential toxic effects of MM54 *in vivo*, C57Bl/6J female mice were administered 2 mg/kg of MM54 or DMSO vehicle, bi-weekly for 4 weeks. At sacrifice, blood was taken for analysis and the heart, kidney, aorta and liver removed, weighed and fixed for histological analysis (as shown in Figure S4). n=4 mice/group, mean±SEM. M: 10^6, K: 10^3.
